# Oridonin Attenuates Aβ_1–42_-Induced Neuroinflammation and Inhibits NF-κB Pathway

**DOI:** 10.1371/journal.pone.0104745

**Published:** 2014-08-14

**Authors:** Sulei Wang, Hui Yang, Linjie Yu, Jiali Jin, Lai Qian, Hui Zhao, Yun Xu, Xiaolei Zhu

**Affiliations:** 1 Department of Neurology, Nanjing Drum Tower Hospital Clinical College of Traditional Chinese and Western Medicine, Nanjing University of Chinese Medicine, Nanjing, P. R. China; 2 Department of Neurology, Affiliated Drum Tower Hospital of Nanjing University Medical School, Nanjing, P. R. China; Massachusetts General Hospital, United States of America

## Abstract

Neuroinflammation induced by beta-amyloid (Aβ) plays a critical role in the pathogenesis of Alzheimer’s disease (AD), and inhibiting Aβ-induced neuroinflammation serves as a potential strategy for the treatment of AD. Oridonin (Ori), a compound of Rabdosia rubescens, has been shown to exert anti-inflammatory effects. In this study, we demonstrated that Ori inhibited glial activation and decreased the release of inflammatory cytokines in the hippocampus of Aβ_1–42_-induced AD mice. In addition, Ori inhibited the NF-κB pathway and Aβ_1–42_-induced apoptosis. Furthermore, Ori could attenuate memory deficits in Aβ_1–42_-induced AD mice. In conclusion, our study demonstrated that Ori inhibited the neuroinflammation and attenuated memory deficits induced by Aβ_1–42_, suggesting that Ori might be a promising candidate for AD treatment.

## Introduction

Alzheimer’s disease (AD), as the major cause of dementia, is an irreversible neurodegenerative disorder with progressive cognitive dysfunction, memory impairment and behavioral damage. According to the World Alzheimer Report 2012, there are 36 million people suffering from dementia worldwide in 2010 [1]. The pathological features of AD compose of beta-amyloid (Aβ) plaques (accumulation of extracellular Aβ) and neurofibrillary tangles (NFTs, deposition of intracellular hyperphosphorylated tau protein) [2], [3]. Although the exact mechanism of AD still remains unclear, evidence from experimental models and human brain studies indicates that Aβ-mediated neuroinflammation is associated with the development of AD [4], [5]. The clinical trials also demonstrate that the levels of pro-inflammatory cytokines are significantly increased in the cerebrospinal fluid (CSF) of AD patients [6], and anti-inflammatory drugs including nonsteroidals have shown great benefits in the treatment of AD [7].

Emerging evidence suggests that Aβ plays important roles in the induction of neuroinflammation in AD [8]. The depositions of Aβ activates the astrocytes and microglia [9], which would release a series of proinflammatory and cytotoxic factors, such as inducible nitric oxide synthase (iNOS), interleukin-1β (IL-1β), cyclooxygenase-2 (COX-2) and tumor necrosis factor alpha (TNF-α) [10]. These cytokines contribute to neuronal damage and eventually cellular death [11]. Nuclear factor-κB (NF-κB) regulates the process of inflammation by targeting the downstream genes, such as TNF-α, IL-1β, iNOS, and COX-2 [12]. Reports have indicated that activation of NF-κB pathway, which subsequently results in the secretion of proinflammatory cytokines, is involved in the degeneration of neurons in AD patients [13], [14]. Inhibition of NF-κB signaling can ameliorate neurodegeneration and memory impairment [15]. These results indicate that suppression of neuroinflammation and NF-κB pathway may be a beneficial therapy for AD.

Oridonin (Ori), a diterpenoid originated from Chinese herb of Rabdosia rubescens, exhibits a diverse of biological activities, such as anti-inflammation, anti-tumor and anti-oxidation [16]–[18]. Recent studies have demonstrated that Ori inhibits the release of proinflammatory mediators through modulating the functions of microglia [19]. In addition, Ori inhibits the NF-κB activity in TNF-α-induced HepG2 cells [20]. In the current study, we investigated whether Ori could inhibit Aβ_1–42_–induced inflammation and attenuate memory deficits in Aβ_1–42_ induced AD mice.

## Materials and Methods

### Aβ_1–42_ induced AD mice model and Ori treatment

The Aβ_1–42_ (Millipore, CA, USA) was dissolved in 1% NH_3_·H_2_O at a concentration of 1 µg/µl and incubated at 37°C for 5 days to allow for fibril formation. Ori (Chengdu Must Bio-Technology Co., Ltd, Sichuan, China, purity more than 98% measured by reverse phase high-performance liquid chromatography) was dissolved in DMSO at a concentration of 20 mg/ml and diluted to the desired concentration in saline. Aβ_1–42_ (4 µg) was injected into the bilateral hippocampus of male C57BL/6 (B6) mice by infusion cannulae as described previously [21]. Mice were divided into three groups: the control mice with saline, Aβ_1–42_-induced AD mice with saline, and Aβ_1–42_-induced AD mice with Ori (10 mg/kg/day, i.p. for 15 days). Our preliminary data has shown that Ori did not attenuate the memory impairment in Aβ_1–42_-induced AD mice at higher dose (20–50 mg/kg/day) (data not shown). After Ori treatment, the mice were trained and tested in Morris water maze for 6 days, and then sacrificed for the following experiments. All experimental procedures were approved by Animal Care Committee of Nanjing University.

### Real-time PCR

As described previously [22], total RNA of the hippocampus was isolated using Trizol (Invitrogen, USA) and reversed transcribed to cDNA using a reverse transcriptase kit (Takara, Dalian, China). Quantitative PCR was performed using ABI 7500 system (Applied Biosystems, USA) by a SYBR green kit (Takara, Dalian, China). The primers (Invitrogen, USA) are as follows:

IL-1β: F: AAGCCTCGTGCTGTCGGACC, R: TGAGGCCCAAGGCCACAGGT;

IL-6: F: GCTGGTGACAACCACGGCCT, R: AGCCTCCGACTTGTGAAGTGGT;

IL-10: F: GGCATGAGGATCAGCAGGGGC, R: TGGCTGAAGGCAGTCCGCAG;

iNOS: F: CAGCTGGGCTGTACAAACCTT, R: CATTGGAAGTGAAGCGTTTCG;

COX-2: F: GATGACTGCCCAACTCCC, R: AACCCAGGTCCTCGCTTA;

TNF-α: F: CAAGGGACAAGGCTGCCCCG, R: GCAGGGGCTCTTGACGGCAG;

MCP-1: F: CCAGCACCAGCACCAGCCAA, R: TGGATGCTCCAGCCGGCAAC;

GAPDH: F: GCCAAGGCTGTGGGCAAGGT, R: TCTCCAGGCGGCACGTCAGA.

### Western blotting

Western blotting analysis was performed as previously [23]. Briefly, cytoplasmic and nuclear proteins of the hippocampus were collected using cytoplasmic and nuclear protein extraction kit (Thermo, USA) according to the manufacturer’s instruction. The proteins were separated by SDS-PAGE and electrophoretically transferred onto polyvinylidene fluoride membranes. Membranes were blocked with 5% skimmed milk for 1 h and incubated overnight at 4°C with anti-COX-2 (1∶1000, Santa Cruz, USA), anti-iNOS (1∶500, Bioworld, USA), anti-NF-κB p65 (1∶1000, Cell Signaling, USA), anti-IκBα (1∶1000, Cell Signaling, USA), anti-p-IκBα (1∶1000, Cell Signaling, USA), anti-Bcl-2 (1∶2000, Bioworld, USA), anti-Bax (1∶1000, Cell Signaling, USA), anti-cytochrome c (1∶500, Abcam, USA), anti-VDAC1 (1∶500, Bioworld, USA), anti-Histone (1∶1000, Epitomics, USA) or anti-β-actin (1∶5000, Bioworld, USA). β-actin was used as a loading control. Subsequently, the membranes were incubated with the corresponding secondary antibodies and the reaction was visualized with chemiluminescence reagents provided with an ECL kit (Bioworld) and exposed to a film. The intensity of the blots was quantified with densitometry.

### Immunostaining

Mice were anesthetized and transcardially perfused with 0.9% saline, then perfused with paraformaldehyde. The brains were removed and cut into consecutive frozen sections and then incubated overnight with anti-ionized calcium-binding adaptor molecule 1 (Iba1) (1∶400, Abcam, USA) and anti-glial fibrillary acidic protein (GFAP) (1∶100, BD, USA) at 4°C. The secondary antibody (1∶200, Invitrogen) was applied to the sections for 1 h at room temperature. The images were performed by a fluorescent microscope (Olympus, Japan). All images were analyzed by Image J for counting of automatically recognized cells. The means were calculated from 5 randomly selected fields in the hippocampus and 5 consecutive sections were analyzed for each brain. All counting procedures were conducted in a randomized and blinded manner.

### Morris water maze text

The Morris water maze text was prepared as previously described [21]. Briefly, mice were trained to find a platform in an open circular pool 2 cm under the water surface in the middle of one quadrant. Four training trials per day were conducted for five consecutive days. In each trial, the latency to escape onto the platform was recorded up to 1 min. If a mouse could found the platform, it was allowed to remain on the platform for 5 s, and then returned to the home cage. If the mouse failed to find the platform within 1 min, it was picked up and placed on the platform for 10 s, and the latency was recorded for 1 min. On the 6th day, a probe trial was given for memory retention by removing the platform from the pool, and each mouse was allowed to swim freely for 1 min, the numbers of crossings of the platform were recorded. All data were collected using a computerized video system.

### Statistical analysis

The results were expressed as means ± SEM. The data were subjected to statistical analysis using SPSS version 13.0 (SPSS, Chicago, IL, USA). Group differences in the escape latency and swimming distance during the MWM test were analyzed by two-way analysis of variance (ANOVA) with repeated measures followed by Bonferroni multiple comparison test with day and treatment as the sources of variation. All other data were analyzed with a one-way ANOVA followed by Bonferroni’s post hoc. P<0.05 were considered statistically significant.

## Results

### Ori Inhibits the Release of Pro-inflammatory Factors in the Hippocampus of Aβ_1–42_-induced AD Mice

**Figure 1:**
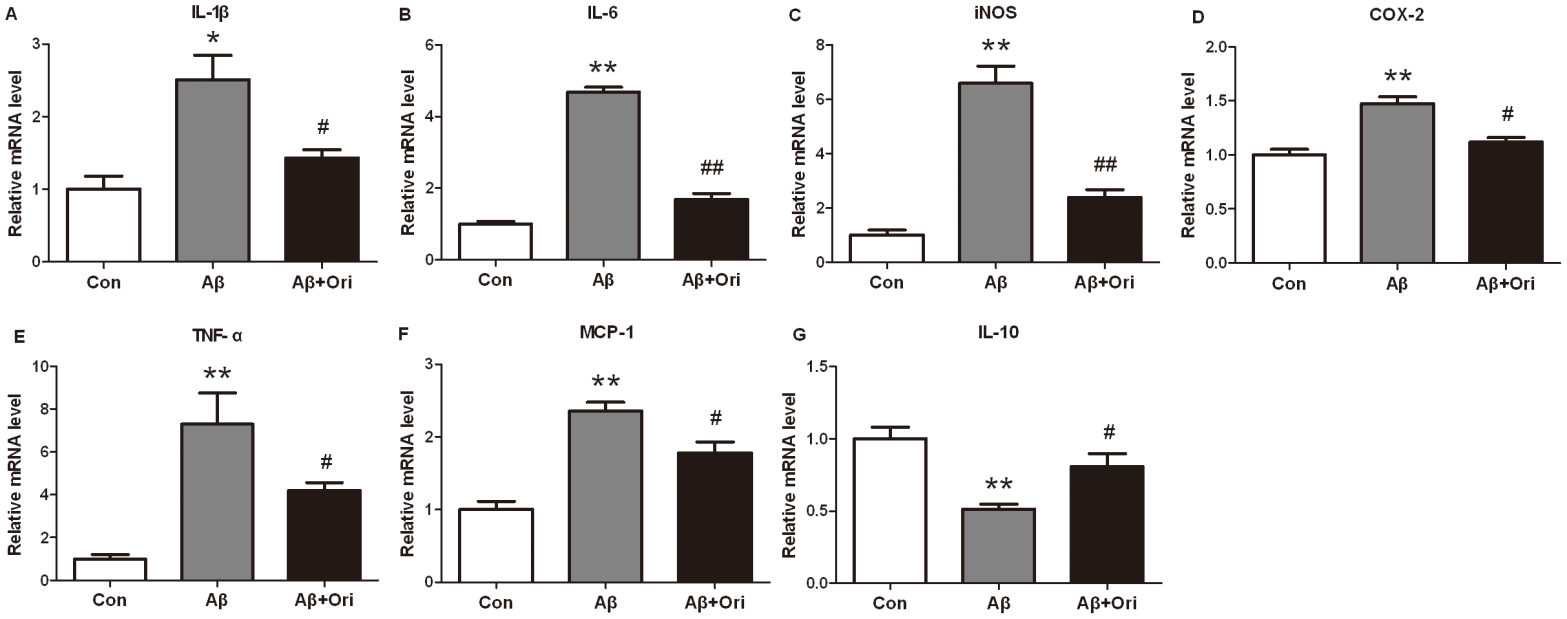
Ori reduces the inflammatory factors in Aβ_1–42_ induced AD mice. The mRNA levels of IL-1β (A), IL-6 (B), iNOS (C), COX-2 (D), TNF-α (E), MCP-1 (F) and IL-10 (G) were measured in each group by Real-time PCR. GAPDH was used as an internal control. n = 6 mice per group. *P<0.05, **P<0.01 vs. control group; #P<0.05, ##P<0.01 vs. Aβ_1–42_ induced AD mice.

**Figure 2:**
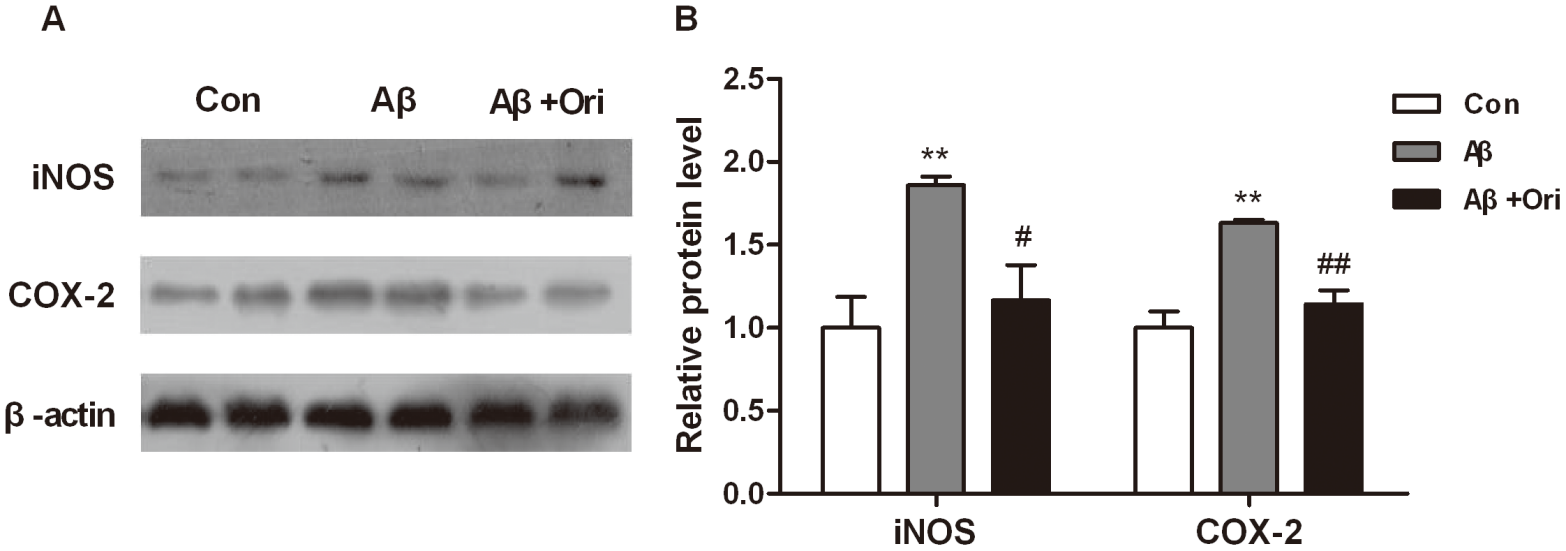
Effects of Ori on the protein levels of iNOS and COX-2 in Aβ_1–42_ induced AD mice. (A) Representative images of western blotting showing Ori inhibited the expression of iNOS and COX-2 in Aβ_1–42_ induced AD mice. (B) Quantitative analysis of iNOS and COX-2 expression. n = 3 mice per group. *P<0.05, **P<0.01 vs. control group; #P<0.05, ##P<0.01 vs. Aβ_1–42_ induced AD mice.

To examine the effects of Ori on Aβ_1–42_-induced inflammation *in vivo*, the relative mRNA levels of IL-1β, IL-6, IL-10, COX-2, iNOS, TNF-α, and MCP-1, and the relative protein levels of iNOS and COX-2 in the hippocampus of mice were tested. The results showed that Aβ_1–42_ could significantly increase the expression of inflammatory factors (IL-1β: P<0.05, IL-6: P<0.01, iNOS: P<0.01, COX-2: P<0.01, TNF-α: P<0.01, MCP-1: P<0.01 versus control group, Figure 1A–F). However, Ori could significantly decrease the levels of these pro-inflammatory cytokines induced by Aβ_1–42_ (IL-1β: P<0.05, IL-6: P<0.01, iNOS: P<0.01, COX-2: P<0.05, TNF-α: P<0.05, MCP-1: P<0.05 versus Aβ group). Meanwhile, the mRNA level of IL-10 was decreased in AD group, and it was upregulated by Ori treatment (Figure 1G). In addition, Ori attenuated the protein levels of iNOS and COX-2 in the hippocampus of AD mice (Figure 2).

### Ori Ameliorates Microglia and Astrocytes Activation in the Hippocampus of AD Mice

**Figure 3:**
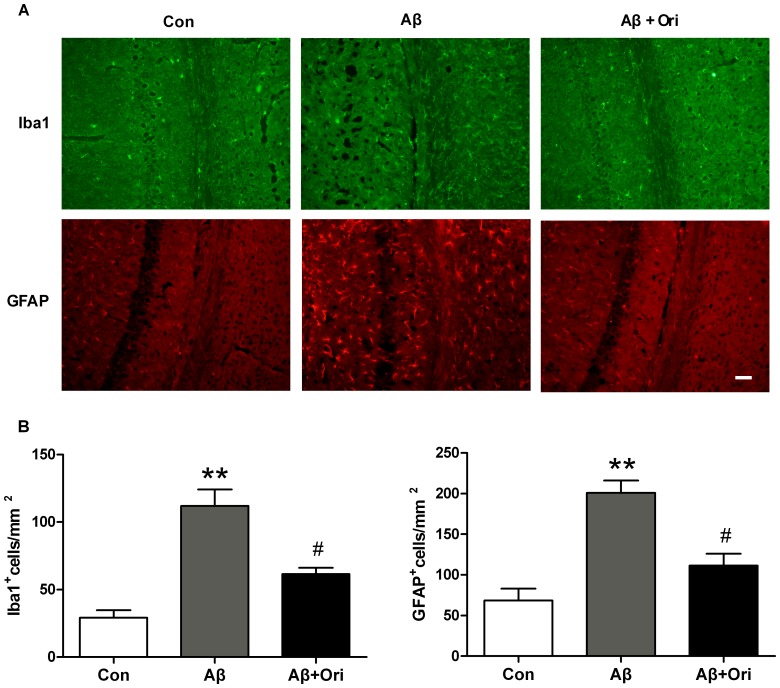
Ori suppresses glial activation in Aβ_1–42_ induced AD mice. (A) Immunostaining for Iba1 and GFAP in the hippocampus of mice. (B) Quantitative analysis of Iba1 and GFAP staining. n = 6 mice per group. Scale bar  = 50 µm. *P<0.05, **P<0.01 vs. control group; #P<0.05, ##P<0.01 vs. Aβ_1–42_ induced AD mice.

To verify whether Ori could inhibit Aβ_1–42_ stimulated activation of microglia and astrocytes, Iba-1 and GFAP staining was performed. Iba-1 and GFAP were specific markers of activated microglia and astrocytes respectively. As shown in Figure 3, the expression of Iba-1 was significantly increased in the hippocampus of Aβ_1–42_-induced AD mice (P<0.01), while treatment with Ori significantly suppressed Iba-1 expression (P<0.05). Ori also reduced the GFAP expression in the hippocampus of AD mice (P<0.05).

### Ori Inhibits Aβ_1–42_-induced Activation of NF-κB p65 Signaling Pathway *in vivo*

**Figure 4:**
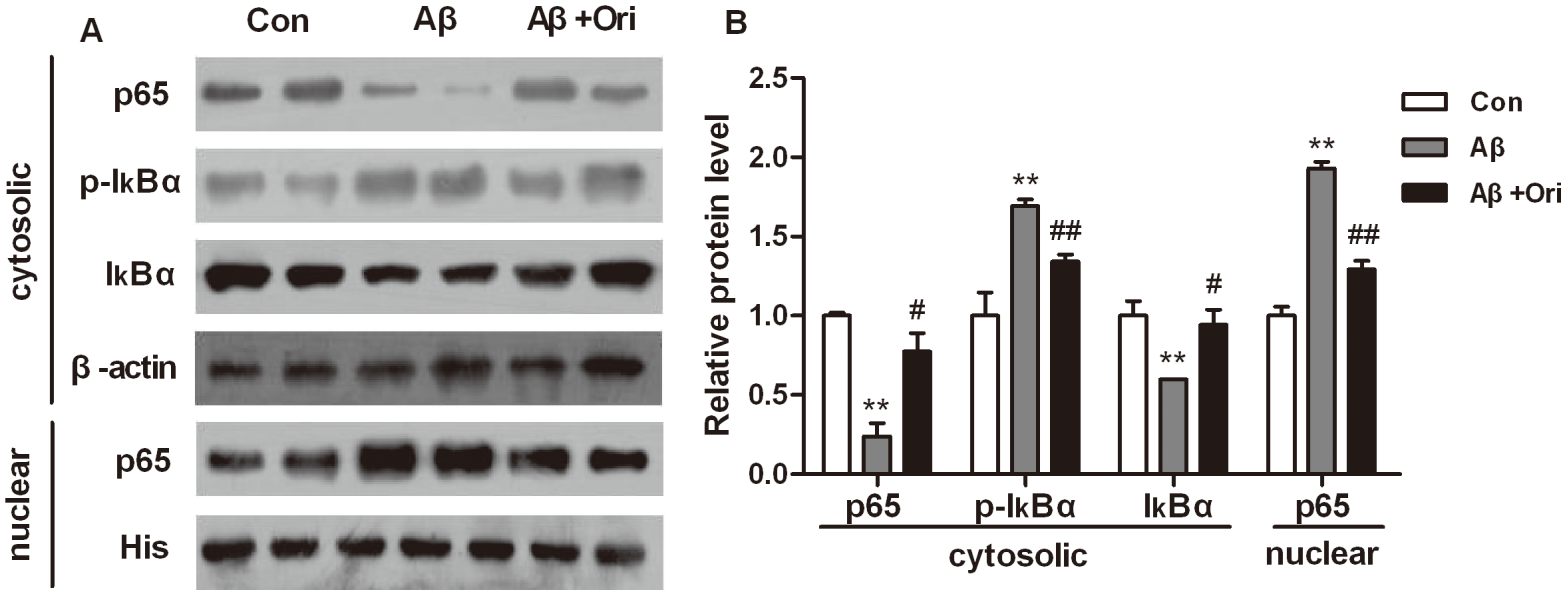
Ori inhibits Aβ_1–42_ induced activation of NF-κB in Aβ_1–42_ induced AD mice. (A) Representative image of western blotting of p65, p-IκBα, IκBα in the cytosolic and p65 in nuclear. (B) Quantitative analysis of Figure 4A. n = 6 mice per group. *P<0.05, **P<0.01 vs. control group; #P<0.05, ##P<0.01 vs. Aβ_1–42_ induced AD mice.

NF-κBp65 signaling pathway is one of the most important pathways in modulating inflammation in AD. Therefore, we explored the role of Ori in Aβ_1–42_-induced activation of NF-κB p65 signaling pathway *in vivo*. As shown in Figure 4, Aβ_1–42_ treatment significantly increased the phosphorylation of IκBα (P<0.01). However, Ori treatment could decreased the phosphorylation of IκBα in the hippocampus of AD mice (P<0.01). In addition, Ori inhibited the Aβ_1–42_-induced degradation of IκBα (P<0.05) and translocation of NF-κB p65 (P<0.01).

### Ori Decreases Mitochondrial Injury in the Hippocampus of AD Mice

**Figure 5:**
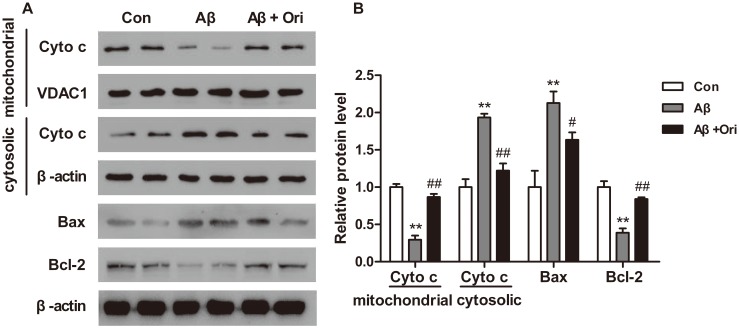
Ori decreases mitochondrial dysfunction in the hippocampus of Aβ_1–42_ induced AD mice. (A) Representative images of western blotting of cytochrome c, Bax and Bcl-2. (B) Quantitative analysis of Figure 5A. n = 6 mice per group. *P<0.05, **P<0.01 vs. control group; #P<0.05, ##P<0.01 vs. Aβ_1–42_ induced AD mice.

Since emerging evidence suggested that Aβ-induced inflammation may contribute to neuronal apoptosis in AD, we investigated the mitochondrial functions in the hippocampus of Ori-treated AD mice. As shown in Figure 5, Ori treatment inhibited the release of cytochrome c from the mitochondria to the cytoplasm (P<0.01). In addition, Aβ_1–42_ increased the expression of Bax (P<0.01) and decreased the expression of Bcl-2 (P<0.01). However, Ori treatment could decrease the level of Bax (P<0.05) and increased the level of Bcl-2 (P<0.01), which indicated that Ori could attenuate the mitochondrial dysfunction induced by Aβ_1–42._

### Ori Improves Cognitive Impairment in Aβ_1–42_-induced AD Mice

**Figure 6:**
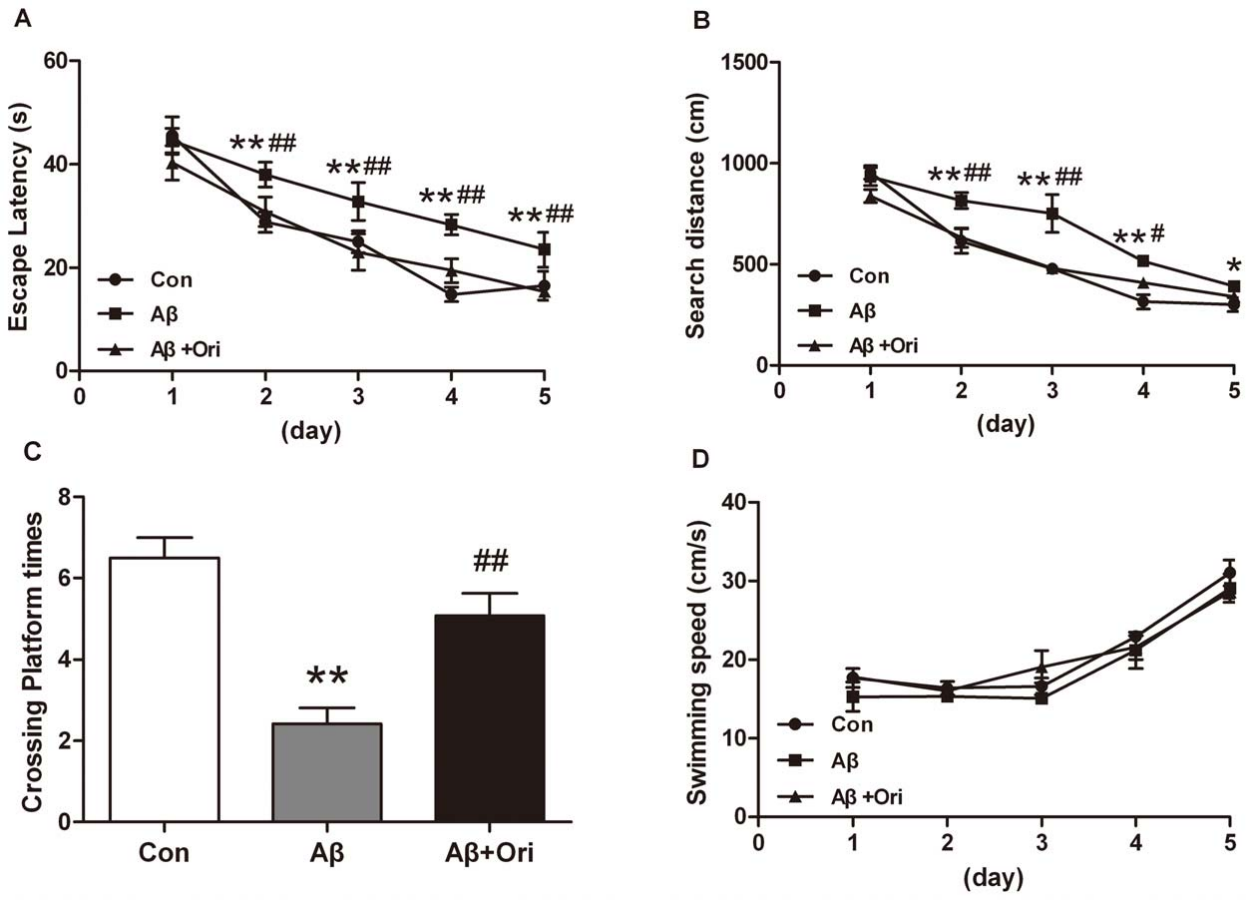
Ori attenuates learning and memory impairment in Aβ_1–42_ induced AD mice in the Morris water maze test. (A) Escape latency for escape to a submerged platform in the training trails. **P<0.01 vs. control group; ##P<0.01 vs. Ori-treated AD mice. (B) Searching distance for escape to a submerged platform in the training trails. **P<0.01 vs. control group; #P<0.05, ##P<0.01 vs. Ori-treated AD mice. (C) Crossing platform times in the probe trail. **P<0.01 vs. control group; ##P<0.01 vs. Aβ_1–42_ induced AD mice. (D) Swimming speed in the training trails. n = 6 mice per group.

To explore whether Ori could improve cognitive impairment in Aβ_1–42_- induced AD mice, Morris water maze test was performed. As shown in Figure 6A, the mean escape latency of Aβ_1–42_ induced AD mice was significantly increased compared with control group (P<0.01), while Ori-treated AD mice showed significant improvements compared with AD mice after the training periods (P<0.01). The searching distance by the Ori-treated AD mice was significantly decreased compared with that of AD mice (P<0.01, Figure 6B). Moreover, on the 6th day, the platform was removed and the probe trail was conducted. The number of platform crossings by the Ori-treated AD mice was significantly higher than that of AD mice (P<0.01, Figure 6C). In addition, there were no differences for swimming speed among these three groups (Figure 6D).

## Discussion

AD is characterized by neurodegeneration and is the most common type of dementia. The Aβ deposition and formation of plaques in the brain have been thought as key events during the progression of AD [24]. In the present study, we demonstrated that Ori attenuated memory impairment in Aβ_1–42_-induced AD mice. Furthermore, Ori could suppress the inflammatory response, and the underlying mechanism might be associated with the inhibition of NF-κB pathway.

Growing data from basic and clinical studies indicate that inflammation induced by Aβ is involved in neuronal degeneration in AD [25], [26]. It has been reported that the levels of proinflammatory cytokines are significantly elevated in brains of AD patients, which suggests that inflammation might contribute to the pathogenesis of AD [27]. The deposition of Aβ can activate glial cells which will release a wide spectrum inflammatory cytokines, such as IL-6 and TNF-α [28]. Aβ-induced overproduction of cytokines could lead to neuronal dysfunction and eventually death in AD. Therefore, inhibiting the activation of glial cells and production of proinflammatory cytokines may contribute to neuroprotection. Previous study indicated that Iba-1 and GFAP was increased in the brain of AD patients [29], [30]. In the present study, the levels of Iba-1 and GFAP were significantly decreased in the Ori-treated mice compared with AD mice, which suggested that Ori suppressed the activation of microglia and astrocytes. Consistently, the results indicated that Ori could inhibit the mRNA levels of IL-1β, IL-6, COX-2, iNOS, TNF-α, and MCP-1 induced by Aβ, and it also up-regulated the expression of IL-10. Collectively, the current findings suggested that Ori modulated inflammatory response by inhibiting the activation of glial cells, which ameliorated the cognitive impairment of Aβ_1–42_-induced AD mice.

NF-κB has been regarded as the key regulator of inflammatory processes, and many studies have showed that suppression of NF-κB pathway ameliorates the neuroinflammation [31], [32]. NF-κB is activated in brains of patients with AD and activated NF-κB is also detected in Aβ surrounding areas [33], [34]. In the resting cells, NF-κB family composed of five members, p65 (RelA), RelB, c-Rel, p50/p105 and p52 which bound to the inhibitory proteins IκB, thereby maintaining NF-κB in an inactive form in the cytoplasm. Upon stimuli, IκB is phosphorylated by IKK (IκB kinase), which then is ubiquitinated and subsequent degraded, leading to translocation of NF-κB to the nucleus and binding to specific target genes, and increased the expression of proinflammatory factors [35]. Hydrogen sulfide inhibits neuroinflammation via suppressing NF-κB pathway and attenuates neuronal death in the hippocampus of Aβ-induced AD rats [36]. Moreover, experimental studies show that suppressing NF-κB could decrease production of Aβ [37], [38]. This study demonstrated that Ori significantly inhibited NF-κB p65 nuclear translocation by attenuating Aβ_1–42_-induced IκBα phosphorylation and degradation *in vivo*.

In summary, the present study shows that Ori, a typical compound of Rabdosia rubescens, attenuates cognitive impairment and inhibits inflammatory response in Aβ_1–42_-induced AD mice. In addition, the anti-inflammatory effects of Ori might be due to inhibiting the NF-κB pathway. These findings suggest that Ori might be a potential agent for AD treatment.
